# Layer-dependent stability of intracortical recordings and neuronal cell loss

**DOI:** 10.3389/fnins.2023.1096097

**Published:** 2023-04-05

**Authors:** Morgan E. Urdaneta, Nicolas G. Kunigk, Jesus D. Peñaloza-Aponte, Seth Currlin, Ian G. Malone, Shelley I. Fried, Kevin J. Otto

**Affiliations:** ^1^Department of Neuroscience, University of Florida, Gainesville, FL, United States; ^2^J. Crayton Pruitt Family Department of Biomedical Engineering, University of Florida, Gainesville, FL, United States; ^3^Department of Electrical & Computer Engineering, University of Florida, Gainesville, FL, United States; ^4^Department of Neurosurgery, Massachusetts General Hospital and Harvard Medical School, Boston, MA, United States; ^5^Boston Veterans Affairs Healthcare System, Boston, MA, United States; ^6^Department of Materials Science and Engineering, University of Florida, Gainesville, FL, United States; ^7^Department of Neurology, University of Florida, Gainesville, FL, United States

**Keywords:** BMI, BCI – brain computer Interface, neuroprosthetic device, neuroprosthesis, neurophysiology, cortex, cortex layers, microelectrode

## Abstract

Intracortical recordings can be used to voluntarily control external devices *via* brain-machine interfaces (BMI). Multiple factors, including the foreign body response (FBR), limit the stability of these neural signals over time. Current clinically approved devices consist of multi-electrode arrays with a single electrode site at the tip of each shank, confining the recording interface to a single layer of the cortex. Advancements in manufacturing technology have led to the development of high-density electrodes that can record from multiple layers. However, the long-term stability of neural recordings and the extent of neuronal cell loss around the electrode across different cortical depths have yet to be explored. To answer these questions, we recorded neural signals from rats chronically implanted with a silicon-substrate microelectrode array spanning the layers of the cortex. Our results show the long-term stability of intracortical recordings varies across cortical depth, with electrode sites around L4-L5 having the highest stability. Using machine learning guided segmentation, our novel histological technique, DeepHisto, revealed that the extent of neuronal cell loss varies across cortical layers, with L2/3 and L4 electrodes having the largest area of neuronal cell loss. These findings suggest that interfacing depth plays a major role in the FBR and long-term performance of intracortical neuroprostheses.

## Introduction

Brain-machine interfaces (BMIs) as assistive technologies have the potential to restore motor function to patients with neurological deficits by reading and writing information to the brain (Nicolelis, 2003). For instance, neural recordings from the motor cortex can be used to bypass spinal lesion (Capogrosso et al., 2016) or control a robotic arm with several degrees of freedom (Hochberg et al., 2012). Moreover, a closed-loop (bi-directional) BMI can be established by combining motor cortex recordings with feedback elicited *via* electrical microstimulation (Urdaneta et al., 2017) within the sensory cortex (Flesher et al., 2016).

There are several different methods to interface with the central nervous system for BMI applications; more invasive techniques offer better recording resolution while causing a more obtrusive immune response. These recording methods vary in portability, invasiveness, and selectivity (Buzsáki et al., 2012). Interfacing directly with single neurons or neuronal ensembles offers the highest degree of spatiotemporal resolution and selectivity for BMI applications (Carmena et al., 2003). This type of interfacing requires implantation of microelectrodes within the cerebral cortex. However, implanting neuroprosthetic devices presents various risks and challenges (Prasad et al., 2014). Post-mortem analysis has shown a foreign body response (FBR) in brain tissue surrounding implanted electrodes (Biran et al., 2005; Esquibel et al., 2020). This FBR is characterized by an activation of local microglia and the formation of an astrocytic glial sheath (Salatino et al., 2017). Moreover, chronically implanted electrodes have shown a loss of neurons in cortical tissue surrounding the electrode (Biran et al., 2005). The extent of neuronal cell loss has been associated with a decrease in recording performance (Salatino et al., 2017) and is exacerbated by longer implantation periods (Biran et al., 2005). However, the profile of neuronal cell loss across cortical depth remains unknown.

Studies across species demonstrate a high degree of variability in the long-term stability of intracortical recordings, potentially hindering the translatability of BMIs. Although some report stable neurons multiple years after implantation (Krüger et al., 2010; Schwarz et al., 2014; Hughes et al., 2021), a larger proportion of studies have shown a substantial decrease in recording performance metrics such as signal-to-noise ratio (SNR), waveform amplitude, and number of active electrode sites occurring within a few weeks post-implantation (Williams et al., 1999; Prasad and Sanchez, 2012; Barrese et al., 2016; Lee et al., 2017; Eisinger et al., 2018). This type of chronic instability represents a challenge for the wide implementation of BMI technologies.

The cerebral cortex is organized in vertical functional units called cortical columns that vary in cellular composition across depth (Mountcastle, 1998). Typically, electrodes are implanted perpendicular to the cortical surface and sit within one or multiple columns depending on the size and orientation of the implant (Rousche and Normann, 1998). Cells in the cortex are densely packed; for example, 1 mm^3^ of mouse cortex contains approximately 200,000 neurons (Braitenberg and Schüz, 2013). However, the number of neurons as well as their interconnections vary substantially across cortical depth in specialized horizontal subdivisions called cortical layers (Hubel and Wiesel, 1968). Each cortical layer presents cell types with unique genetic, functional, and structural features (Gouwens et al., 2019). This gives rise to distinctive morphological and electrophysiological properties across cortical depth (Staining, 2015; Gouwens et al., 2019; Senzai et al., 2019).

Current clinical intracortical device implementations can lead to variations in interfacing cortical depths across electrodes. The only FDA-cleared microelectrode for BMI applications to-date is the Utah array (Rousche and Normann, 1998). This microelectrode array comprises a grid of 10 × 10 of electrodes separated by 400 μm, with the four corner electrodes serving as references. Each shank has a length of 1,500 μm with the recording interface at the tip, as these arrays were initially designed to interface with layer 4 of the primary visual cortex (Rousche and Normann, 1998). Nevertheless, laminar volume variations within the same cortical region (Wagstyl et al., 2020) as well as the non-planar geometry of the cortical surface can cause a significant fraction of array electrode sites to be outside of their targeted depth (Urdaneta et al., 2021). Although the Utah array was originally designed to be implanted in V1, it has been clinically implanted in the primary motor (Hochberg et al., 2012) and somatosensory cortex (Flesher et al., 2016). Even though the canonical laminar circuitry of the cortex is conserved across different cortical areas (Hawkins and Blakeslee, 2007), differences in laminar volume (Wagstyl et al., 2020) and subjects (Hedman et al., 2012) can influence the cortical layer in which the interfacing tip will rest. Given the differences in cytoarchitecture (Staining, 2015; Gouwens et al., 2019) and electrophysiology (Senzai et al., 2019) across cortical layers, the depth of the interfacing electrode may play a previously unexplored role in the long-term stability of intracortical neuroprostheses. Furthermore, cellular response to implants may vary between cortical layers, potentially leading to a depth-dependent FBR.

Here, we implanted adult rats with a single-shank silicon microelectrode device containing electrode sites spanning all the layers of the primary somatosensory cortex. Intracortical recordings and impedance spectroscopy measurements were obtained for 16 weeks post implantation (WPI). Metrics for recordings stability such as SNR, waveform amplitude, and number of active sites were analyzed across cortical depth and time. Moreover, to analyze the extent of neuronal loss surrounding chronically implanted electrodes by cortical depth, we developed a technique called DeepHisto. This method consists of systematically sectioning and staining histological sections across cortical depth and quantifying different metrics *via* machine learning guided segmentation and area estimation. We found that spike amplitude in S1 varies across cortical depth and the long-term stability of intracortical recordings decays in a layer-specific manner. Furthermore, our histological analysis revealed that the extent of neuronal cell loss around the electrode is depth-dependent. Similarly, the degree of morphological distortion and variability of surviving neurons changed based on their proximity to the electrode. Altogether, these findings suggest that interfacing depth plays a pivotal role in the long-term performance of intracortical neuroprostheses.

## Results

**Figure 1:**
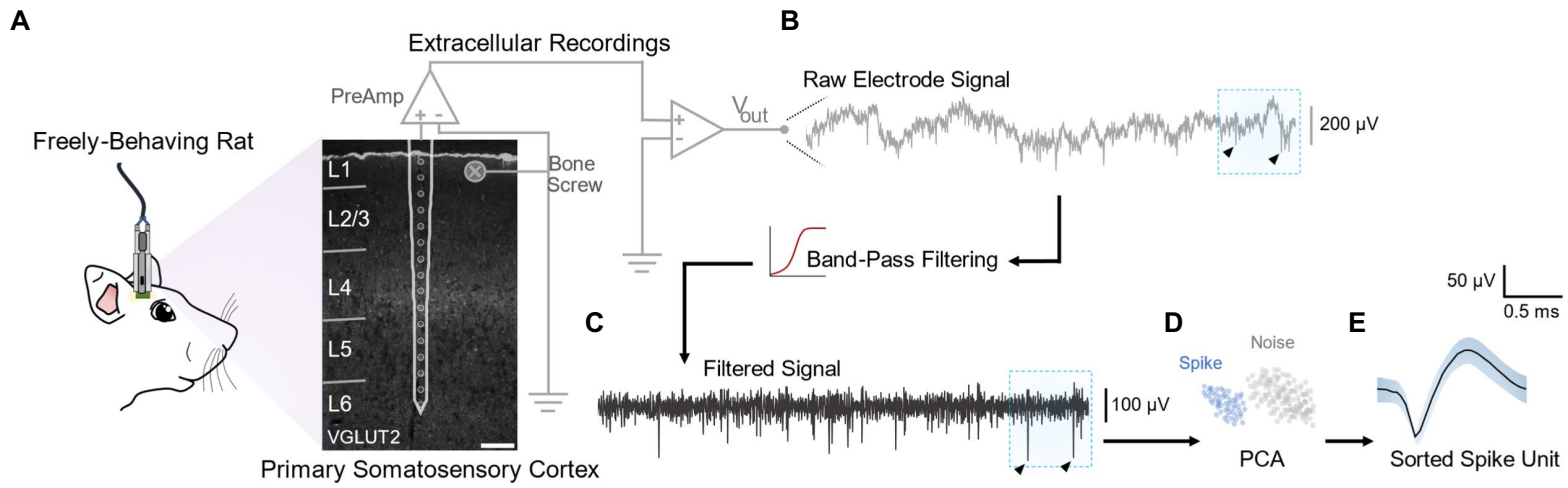
Extracellular recordings from freely behaving animals and signal processing pipeline. **(A)** Extracellular recordings were obtained from planar microelectrode array chronically implanted in the primary somatosensory cortex of freely behaving rats. The 16 channels of the device spanned all cortical layers, as confirmed *via* coronal histological sections of S1 stained with VGLUT2. The electrode was grounded to a bone-screw and signals were pre-amplified through a digital headstage connected to a NeuroDigitizer biological amplifier. **(B)** For each recording channel, voltage traces from raw extracellular recordings were obtained at a sampling frequency of ~24 kHz. **(C)** These raw traces were band-pass filtered (250 Hz −3 kHz). Arrows on raw and filtered traces indicate exemplar downward deflections characteristic of extracellular spikes. **(D)** Spike units were sorted from the filtered signal using principal component analysis (PCA) and k-means clustering using commercially available software. **(E)** Shaded area around the sorted spike waveform represents ± SEM.

Six adult Sprague–Dawley rats were chronically implanted with a silicon microelectrode in the primary somatosensory cortex. The microelectrode device had 16 equidistant electrode sites (Figure 1A) spanning all cortical layers (Supplementary Figure S1). Five-min neural recording sessions were captured at regular intervals from freely behaving animals over a period of 16 weeks and spike sorted using commercially available software (see Methods) (Figures 1B-E).

### Cortical layers 4 and 5 exhibit the highest spike amplitudes

**Figure 2:**
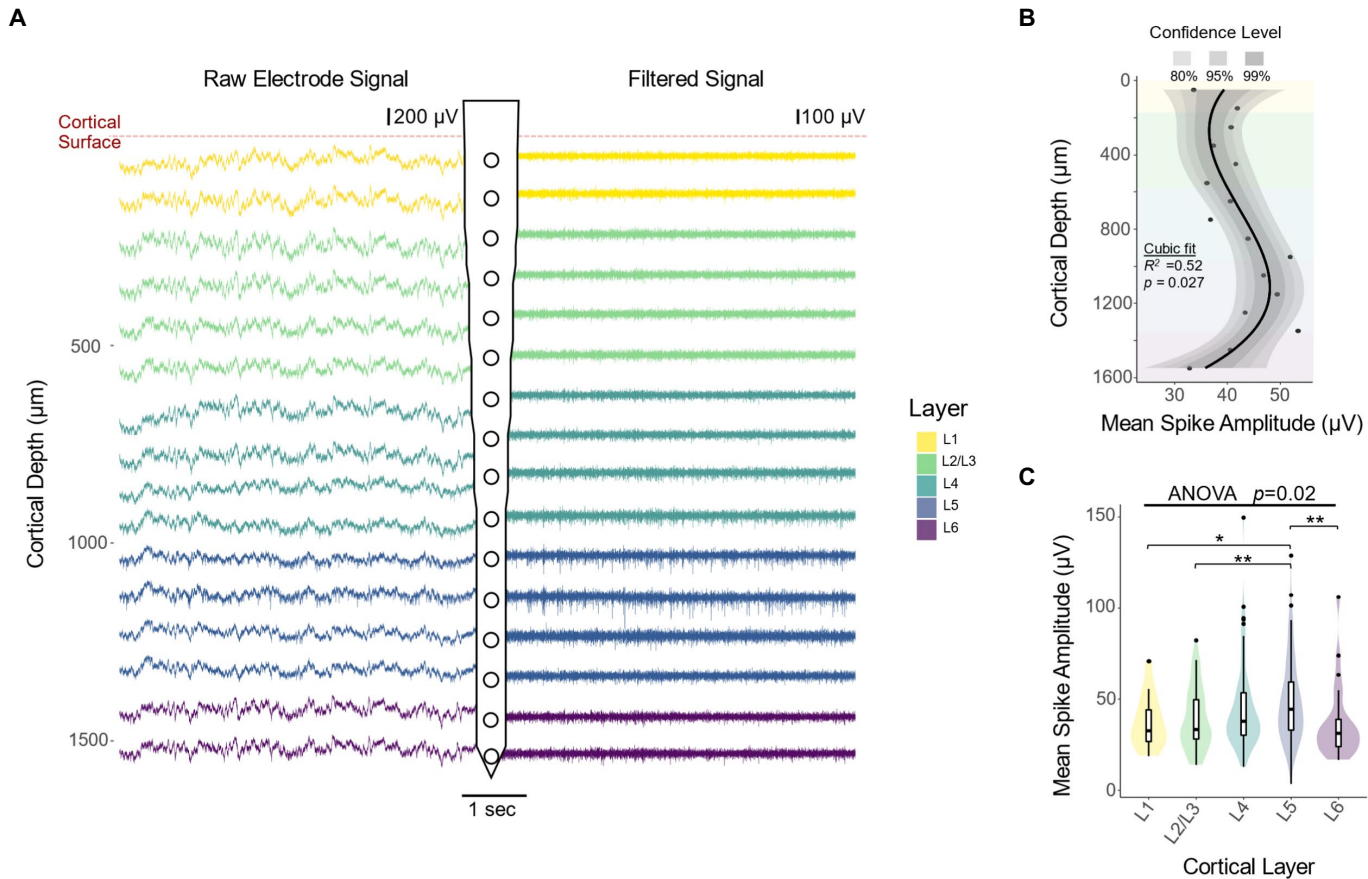
Extracellular recordings across cortical depth. **(A)** Representative raw and filtered recording traces from electrode sites at different cortical depths. Compared to superficial channels, electrode sites located near L4 and L5 contain more high amplitude downward deflections, characteristic of action potentials. **(B)** Mean spike amplitude over depth during the first 2 weeks of implantation reveals an increase in spike amplitude with cortical depth, reaching a maximum approximately 1,100 μm from the cortical surface (*N* = 6). The black line represents a locally fitted polynomial regression of the means and shaded areas represent confidence intervals. **(C)** Quantification of spike amplitude across cortical layers (one-way ANOVA followed by Dunn–Bonferroni’s correction) and pairwise comparisons between layers (two-tailed *t*-test followed by Dunn–Bonferroni’s correction). *p ≤ 0.05, **p ≤ 0.01.

We first investigated how the amplitude of neural recordings vary across S1 cortical layers. Figure 2A shows representative raw (left) and filtered (right) recording traces from each electrode along the microelectrode shank. Compared to superficial channels, electrode sites located near L4 and L5 contain more high amplitude downward deflections, characteristic of action potentials (representative action potentials are shown with black arrows in Figure 1B). To quantify amplitude changes in the raw and filtered data, we spike sorted recording sessions during the first 2 WPI of each animal and calculated the mean amplitude of the resulting waveforms. Our results showed that spike amplitude increases as a function of cortical depth down to L5 from the cortical surface (Figure 2B: 13.9 μV/mm, linear regression slope from 0 to 1,050 μm). This is followed by a decrease in spike amplitude as a function of cortical depth down to L6 (Figure 2B: −31.6 μV/mm, linear regression slope from 1,050 to 1,550 μm). Quantification of the laminar distribution of the spike waveforms over depth showed that spike amplitude varies significantly across cortical layers (Figure 2C: *F*(4,253) = 2.912, *p =* 0.0221; one-way ANOVA). In particular, L5 spikes had higher waveform amplitudes compared to those found in either superficial layers (Figure 2C; L5 vs. L1: *p =* 0.0275; L5 vs. L2/3: *p =* 0.0019; unpaired *t*-test with Dunn–Bonferroni correction) or in the deepest layer (Figure 2C; L5 vs. L6: *p =* 0.0087; unpaired *t*-test with Dunn–Bonferroni correction). These results suggest that interfacing depth can play a role in the ability of electrodes to acquire high amplitude spikes. Moreover, these results provide a foundation from which to study the consistency of these spikes over time.

### The long-term stability of intracortical recordings is layer-dependent

**Figure 3:**
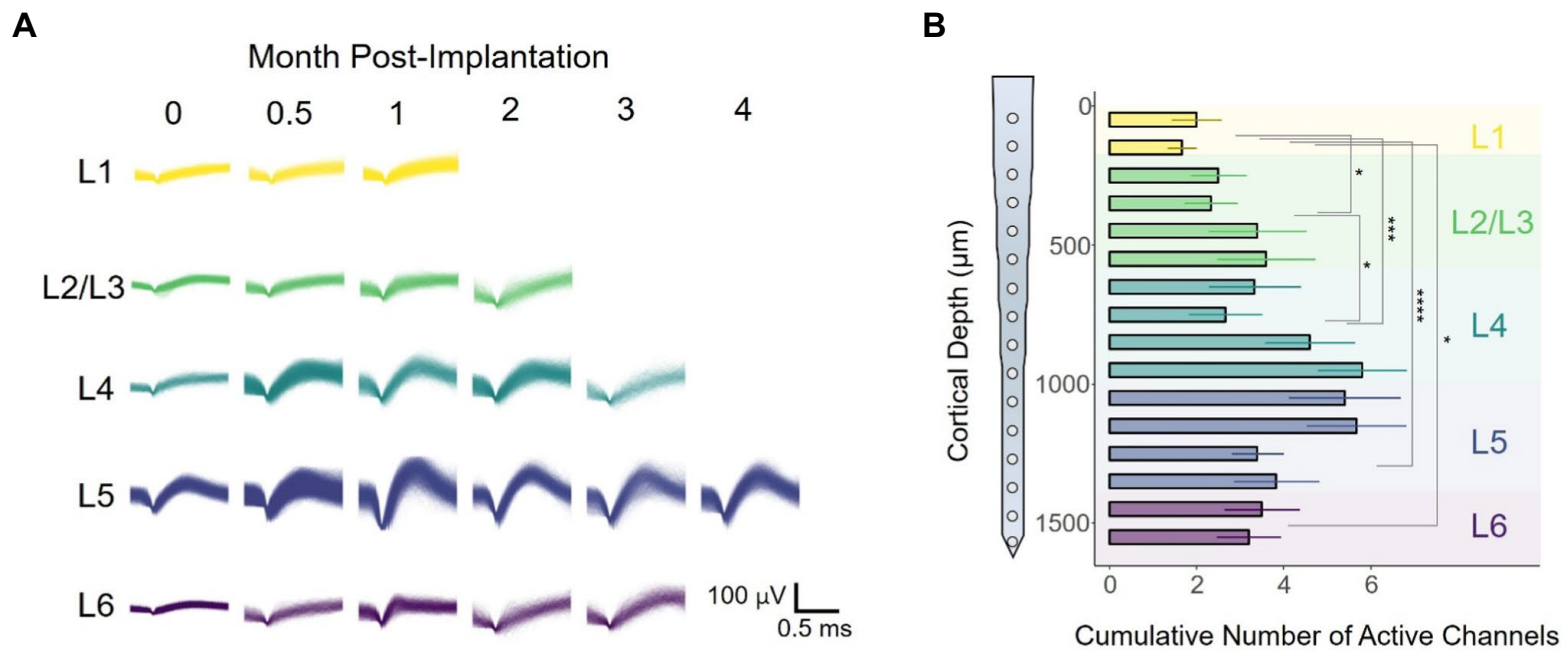
Electrode-sites on layer 4 and 5 remain more active over time. **(A)** Representative spike waveforms of one rat as a function of time. **(B)** Channels in which spikes units were present were deemed as active. Bar chart plot of the average number of active channels per site across the duration of the study. Active channels were binned in cortical layers for quantification (one-way ANOVA followed by Dunn–Bonferroni’s correction). *p ≤ 0.05, **p ≤ 0.01, ***p ≤ 0.001, ****p ≤ 0.0001. Error bars indicate mean ± SEM.

To evaluate whether response stability varies over time post-implantation, we captured intracortical recordings for a period of 16 weeks post-implantation (WPI). Figure 3A shows representative waveforms obtained from one animal across the duration of the study. Each panel contains an overlay of all spikes elicited during a single recording session for each cortical layer. For this particular animal, only recordings from L5 had sortable spike waveforms by the end of the study (Figure 3A). Notably, waveforms obtained from the same channel had different shapes over time. To further quantify this layer-dependent change in recording performance, we calculated the number of active channels (channel with sortable units) each across the duration of the study for each animal. Our results showed significant changes in the cumulative number of active channels across cortical layers (Figure 3B: *F*(4,64) = 3.467, *p =* 0.0117; one-way ANOVA), with the most active sites located around L4 and L5 (Figure 3A; L4 vs. L1: *p =* 0.00084; L5 vs. L1: *p =* 5.9 × 10^−5^; L5 vs. L2/3: *p =* 0.0218; unpaired *t*-test with Dunn–Bonferroni correction). These results indicate that interfacing at a particular layer (i.e., L4-L5) might increase the chances of detecting spikes at chronic time points. Moreover, this trend raises the question of whether these layer-specific changes in long-term recording performance affect other metrics of recording performance such as spike amplitude or SNR.

### Spike waveform amplitude remains stable over time

**Figure 4:**
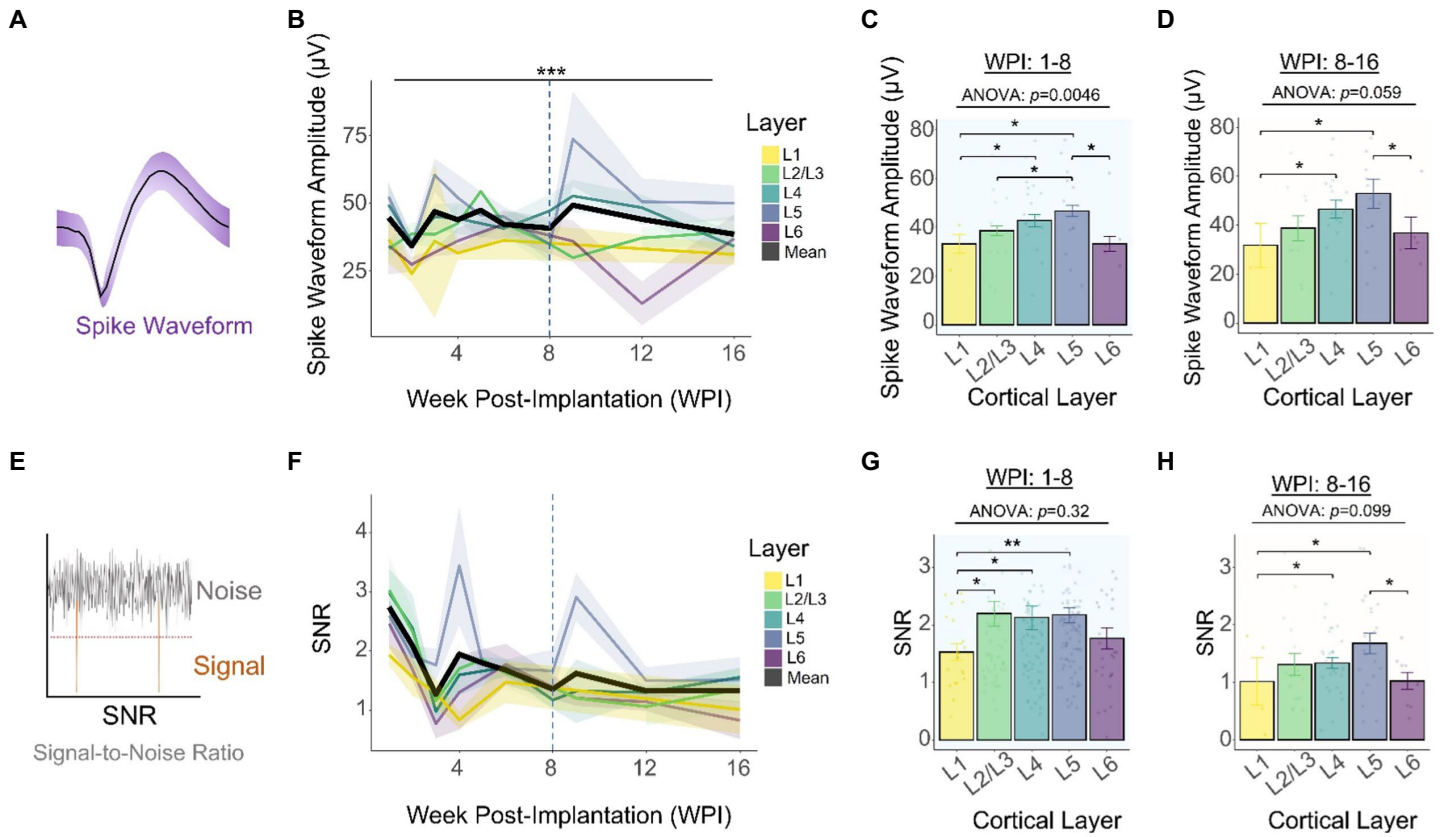
Chronic waveform amplitude and SNR stability across cortical layers. **(A)** Graphical representation of a spike waveform post-sorting. **(B)** Spike waveform amplitude across layers throughout the duration of the study. Top horizontal line represents an analysis of covariance (ANCOVA). To assess time-specific differences in spike amplitude across layers, the spike amplitudes of the **(C)** first (1–8 WPI) and **(D)** second (8–16 WPI) halves of the study were examined. **(E)** SNR was calculated by dividing the peak-to-peak amplitude over two times the noise amplitude (Eq. 2). **(F)** SNR across layers over time. Laminar quantification of SNR during the first **(G)** and second **(H)** half of the study. Analysis of variance and pairwise comparisons between layers **(C, D, G, H)** were performed with a one-way ANOVA and an unpaired *t*-test with Dunn–Bonferroni’s correction, respectively. *p ≤ 0.05, **p ≤ 0.01, ***p ≤ 0.001. Shaded areas and error bars indicate mean ± SEM. (*N* = 6).

We found that the slope of the average waveform amplitude (Figure 4A) across all layers remained constant for the duration of the study (Figure 4B: −0.003 μV/month, linear regression slope). Consistent with our previous observations (Figure 3A), the average spike amplitude increased 12.8% during the first 3 WPI (Figure 4B). Using time as a covariate, we found a statistically significant difference in spike amplitude across layers [Figure 4B: *F*(4,354) = 5.849, *p =* 0.00014, ANCOVA interaction]. To further elucidate if variations in amplitude are different across layers, we compared spike amplitudes across depth during the first (8 WPI) and second (8–16 WPI) halves of our study. Our analysis showed that spike amplitude varies across layers during the first (Figure 4C: *F*(4,277) = 3.849, *p =* 0.0462; one-way ANOVA) and second half of the study (Figure 4D: *F*(4,77) = 2.374, *p =* 0.0594; one-way ANOVA). Spike amplitude was the highest in L4 and L5 for the first (Figure 4C; L1 vs. L2/3: *p =* 0.012; L1 vs. L4: *p =* 0.002; L1 vs. L5: *p =* 0.0021; L5 vs. L2/3: *p* = 0.0091; unpaired *t*-test with Dunn–Bonferroni correction) and second half of the study (Figure 4D; L5 vs. L6: *p* = 0.031; L5 vs. L1: *p* = 0.012; L4 vs. L1: *p* = 0.029). Together, these findings indicate that the spike amplitude patterns across cortical layers observed during the initial recording sessions (Figures 2B,C) remained constant over time, with L4 and L5 having the highest spike amplitudes.

### Recording SNR decreases over time in a layer-specific manner

To further investigate the long-term performance of intracortical recordings, the peak-to-peak amplitude was divided over two times the noise level to calculate the signal-to-noise ratio (SNR) (Figure 4E). The average SNR across all layers decreases from 1 WPI (2.74 ± 0.18) to 16 WPI (1.33 ± 0.14), resulting in an average decay of −0.338 SNR units/month (Figure 4F: linear regression slope). Our analysis of covariance showed that this decay in SNR performance did not vary significantly across layers over time [Figure 4F: *F*(4,354) = 1.463, *p =* 0.213; ANCOVA interaction]. Furthermore, to quantify SNR differences between cortical layers, we compared the SNR of the first and second halves of the study. Quantification of the first 8 WPI showed that SNR did not vary significantly across layers (Figure 4G: *F*(4,277) = 1.177, *p =* 0.321; one-way ANOVA). However, L1 had a significantly lower SNR compared to most layers (Figure 4G; L1 vs. L2/3: *p =* 0.0127; L1 vs. L4: *p =* 0.002; L1 vs. L5: *p =* 0.0021; unpaired *t*-test). During the second half of the study, the average SNR across all layers decreased by 34.1% and SNR differences across cortical layers became more pronounced [Figure 4H: *F*(4,77) = 2.023, *p =* 0.0995; one-way ANOVA]. In particular, L5 had the highest SNR (Figure 4H: L5 vs. L6: *p =* 0.043; unpaired *t*-test with Dunn–Bonferroni correction). This evidence indicates that although the average SNR decays over time, it is possible to obtain high SNR signals at chronic timepoints depending on the interfacing layer.

### Impedance peaks on week 1 before flattening

**Figure 5:**
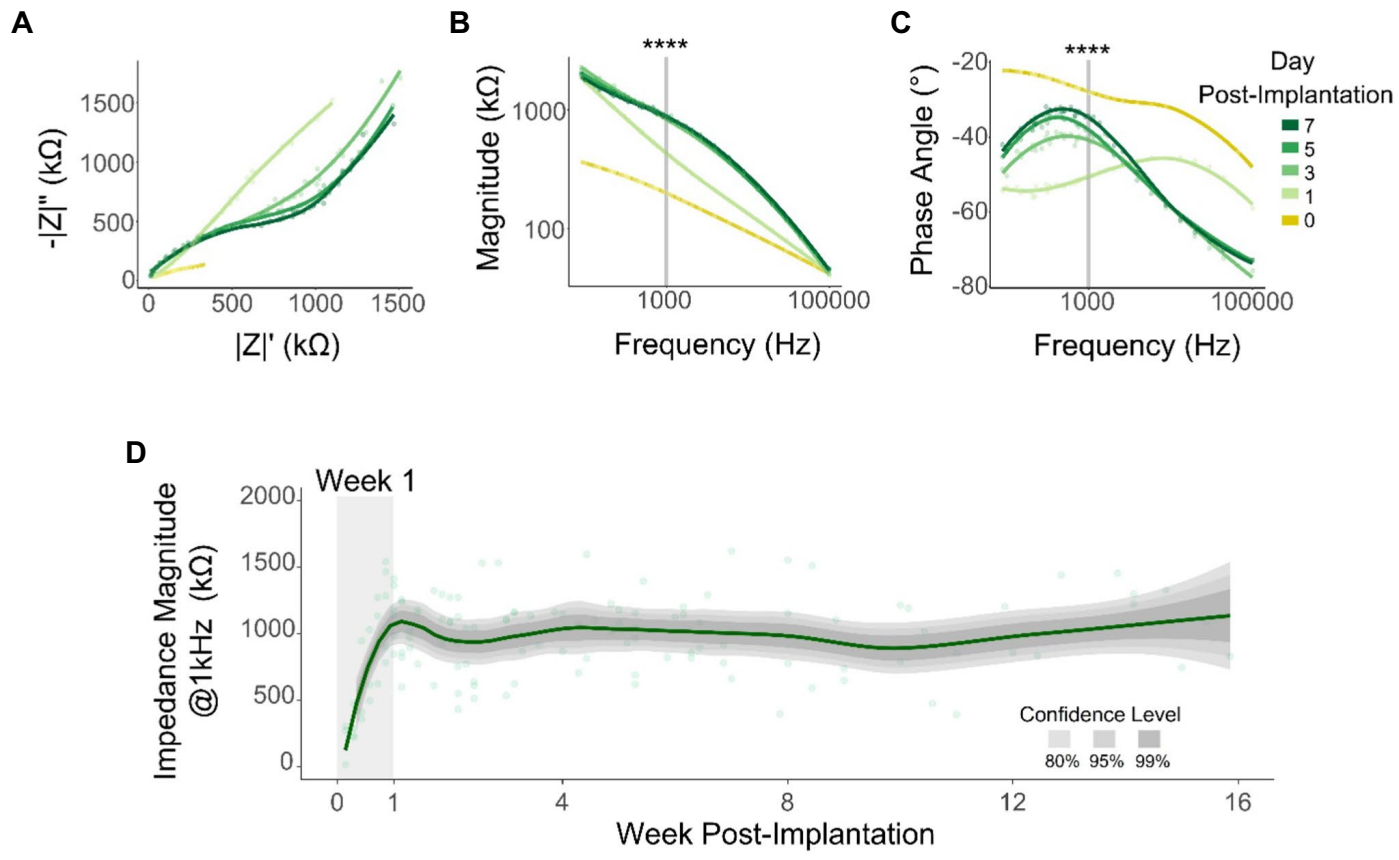
Electrical impedance over time. Representative changes in impedance during the first week post-implantation: Nyquist **(A)**, impedance magnitude **(B)**, and phase angle **(C)**. Shaded lines indicate data points at 1 kHz that were used for statistical comparison (Kruskal–Wallis test) for all tested channels (*n* = 545). **(D)** Mean impedance magnitude at 1 kHz over time. Line represents a locally fitted polynomial regression of the means and shaded areas represent confidence intervals (*N* = 6). ****p ≤ 0.0001.

Previous studies have reported correlations between changes in SNR and changes in electrode impedance (Ludwig et al., 2006). We measured impedance spectroscopy at a logarithmic frequency range (10 Hz to 100 kHz) throughout the duration of the study. By plotting the real vs. imaginary components of the impedance for all tested frequencies (Nyquist plot) during the first WPI, we observed an increase in both resistance and reactance (Figure 5A). Similarly, our results showed inter-day differences that stabilize after day 1 in both impedance magnitude (Figure 5B) and phase angle (Figure 5C) across the range of frequencies tested here. To quantify these differences, we compared the magnitude and phase angle values of all tested channels (*n* = 545) for all 6 animals at 1 kHz (Figures 5B,C, vertical line). This frequency is related to the fundamental frequency of action potentials and is commonly used to assess recording capabilities (Neto et al., 2018). Consistent with previous reports (Vetter et al., 2004), there were highly significant differences in both impedance magnitude (Figure 5B: χ^2^ = 311.86, *p* < 2.2 × 10^−16^, df = 8; Kruskal–Wallis test) and phase angle (Figure 5C: χ^2^ = 164.72, *p* < 2.2 × 10^–16^, df = 8; Kruskal–Wallis test) during the first WPI. To further assess impedance magnitude beyond the first WPI, we plotted 1 kHz impedance magnitude across the 16-week duration of the study. Figure 5D shows an increase of 480.6% in impedance magnitude from day 1 (195.6 ± 156.9 kΩ) to day 7 (1135.8 ± 854.2 kΩ) (Figure 5D: shaded area). After this initial increase however, impedance magnitude remained approximately constant for the remaining 15 weeks (Figure 5D: −21.7 kΩ/month, linear regression slope from week 2 to week 16). The stability in electrical impedance over weeks 2–16 is in contrast with the decay in SNR and recording performance over this same time period and therefore suggests other factors were likely to underlie the change in performance. Hence, we decided to investigate whether the ability to record neural signals chronically across cortical depth was influenced by neuronal cell loss.

### Neuronal cell loss area changes across depth

**Figure 6:**
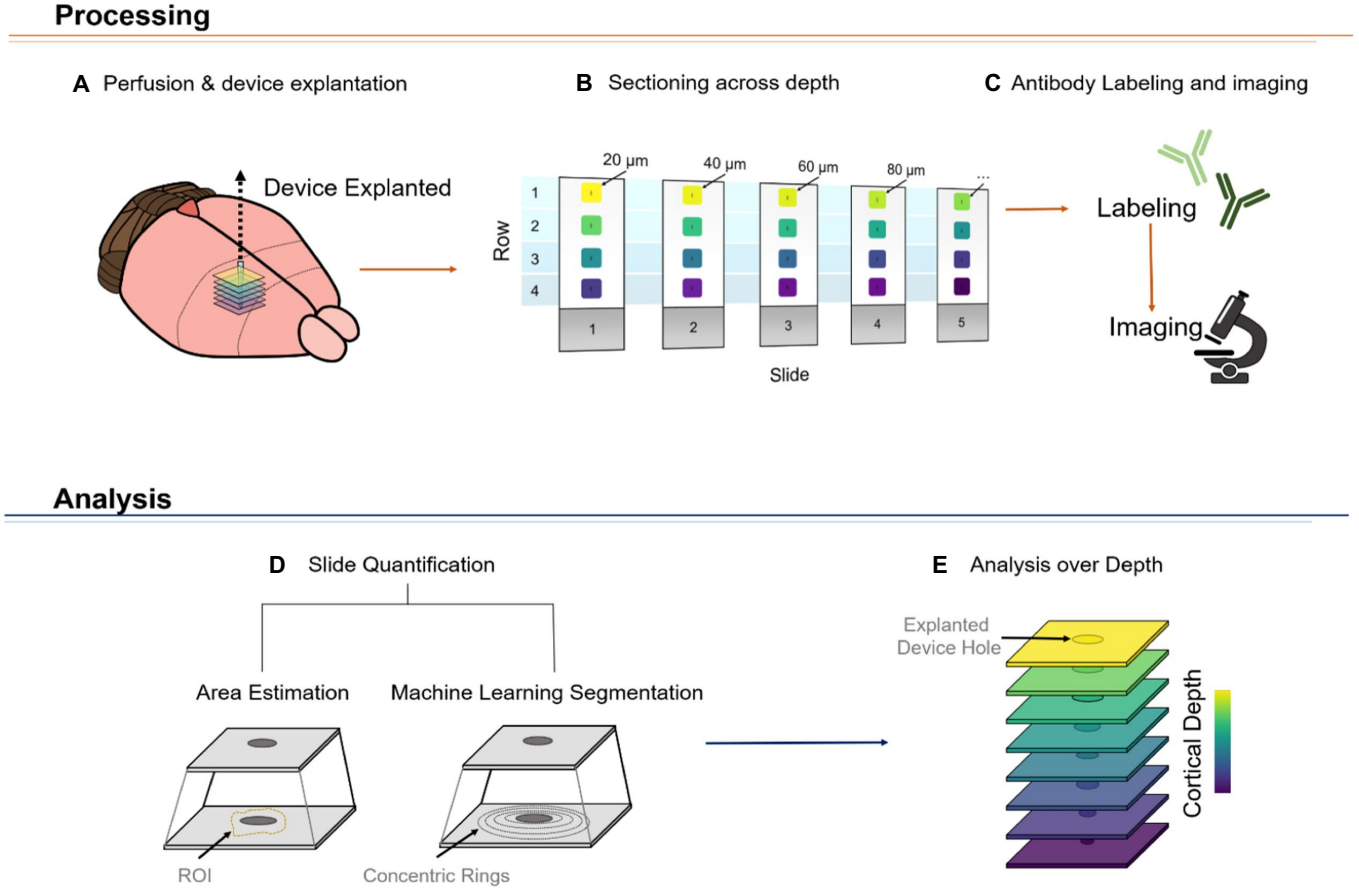
Representative diagram of DeepHisto pipeline. The tissue processing phase consists of carefully removing the silicon microelectrode after perfusion and fixation of the brain with paraformaldehyde **(A)**. The area around the explanted device is isolated and systematically cryosectioned to keep track of the relative depth of each cut **(B)**. The slides are then labeled with antibodies and imaged **(C)**. Subsequently, the tissue response of each imaged slide is quantified **(D)**. This quantification process could be achieved by either estimating the region of interest (ROI) of a specific marker (i.e., delineating the area neuronal cell loss around the electrode) or by obtaining fluorescence intensity levels of concentric rings around the device hole. Lastly, by plotting these values over depth, a depth-specific profile of the tissue response can be obtained **(E)**.

**Figure 7:**
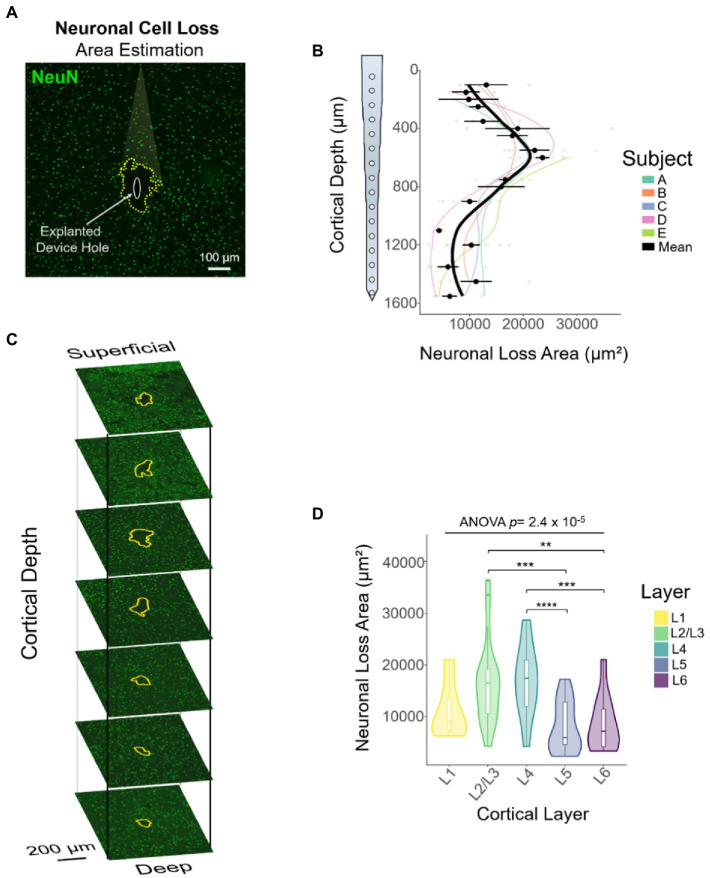
Quantification of neuronal cell loss area across cortical depth using DeepHisto. **(A)** The area of neuronal cell loss was manually assessed by blinded evaluators. Yellow dotted outline represents the estimate of area of neuronal cell loss surrounding the explanted electrode (white oval). **(B)** Representative DeepHisto stacks of cortical slice histological analysis across cortical depth for one animal. Yellow outlines represent neuronal cell loss surrounding the explanted electrode. **(C)** Quantification of neuronal cell loss area across cortical depth. Colored lines represent locally fitted polynomial regressions for each subject and black line represents the mean (*N* = 5). **(D)** Quantification of neuronal cell loss area across cortical layers (one-way ANOVA) and pairwise comparisons between layers (unpaired *t*-test followed by Dunn–Bonferroni’s correction). *p ≤ 0.05, **p ≤ 0.01, ***p ≤ 0.001, **** *p* ≤ 0.0001. Error bars indicate mean ± SEM.

Attempts to evaluate the FBR using device-capture histology (Woolley et al., 2011) have shown a non-uniform microglial response across cortical depth (Woolley et al., 2013). In addition, using standard histological techniques, groups have shown qualitative differences in FBR markers across representative (superficial, middle, and deep) cortical depths (Kozai et al., 2014; Nolta et al., 2015; Rosenfeld et al., 2020). Here, we established a novel method, referred to as DeepHisto, which consisted of systematically sectioning, staining, and quantifying different metrics of neural tissue response surrounding chronically implanted electrodes. We used DeepHisto to determine the extent of neuronal cell loss across cortical depth. Following perfusion and cryosection (Figures 6A,B), histological sections were stained for neuronal nuclei with NeuN and imaged (Figure 6C). The neuronal cell loss area estimation (Figure 6D) was performed by tracing the area surrounding the explanted hole with no evident neuronal bodies for each slice (Figure 7A: yellow outline). A representative histological overlay of one of the animals shows that the extent of neuronal cell loss fluctuates across cortical depth (Figure 7B) and therefore, we quantified the area of neuronal loss across cortical depth for each animal (Figure 7C). The area of neuronal cell loss peaked around 600 μm from the pia, corresponding approximately to the lower portion of L2/3 and the upper portion of L4 (Figure 7C). This peak is followed by a decrease in area among deeper cortical layers. Quantification of the differences as a function of layer revealed that neuronal cell loss changes significantly across cortical layers (Figure 7D: *F*(4,77) = 7.82, *p =* 2.42 × 10^−5^; one-way ANOVA). In particular, the largest areas of cell loss occurred at L2/3 and L4 (Figure 7D: L2/3 vs. L5: *p =* 0.0012; L2/3 vs. L6: *p =* 0.012; L4 vs. L5: *p =* 0.0003; L4 vs. L6: *p =* 0.005, Tukey’s *post hoc* test). These results provide evidence that the extent of neuronal cell loss across cortical depth is non-uniform. However, this finding could be attributed to intrinsic differences in neuronal density across depth.

### Neuronal density and morphology

**Figure 8:**
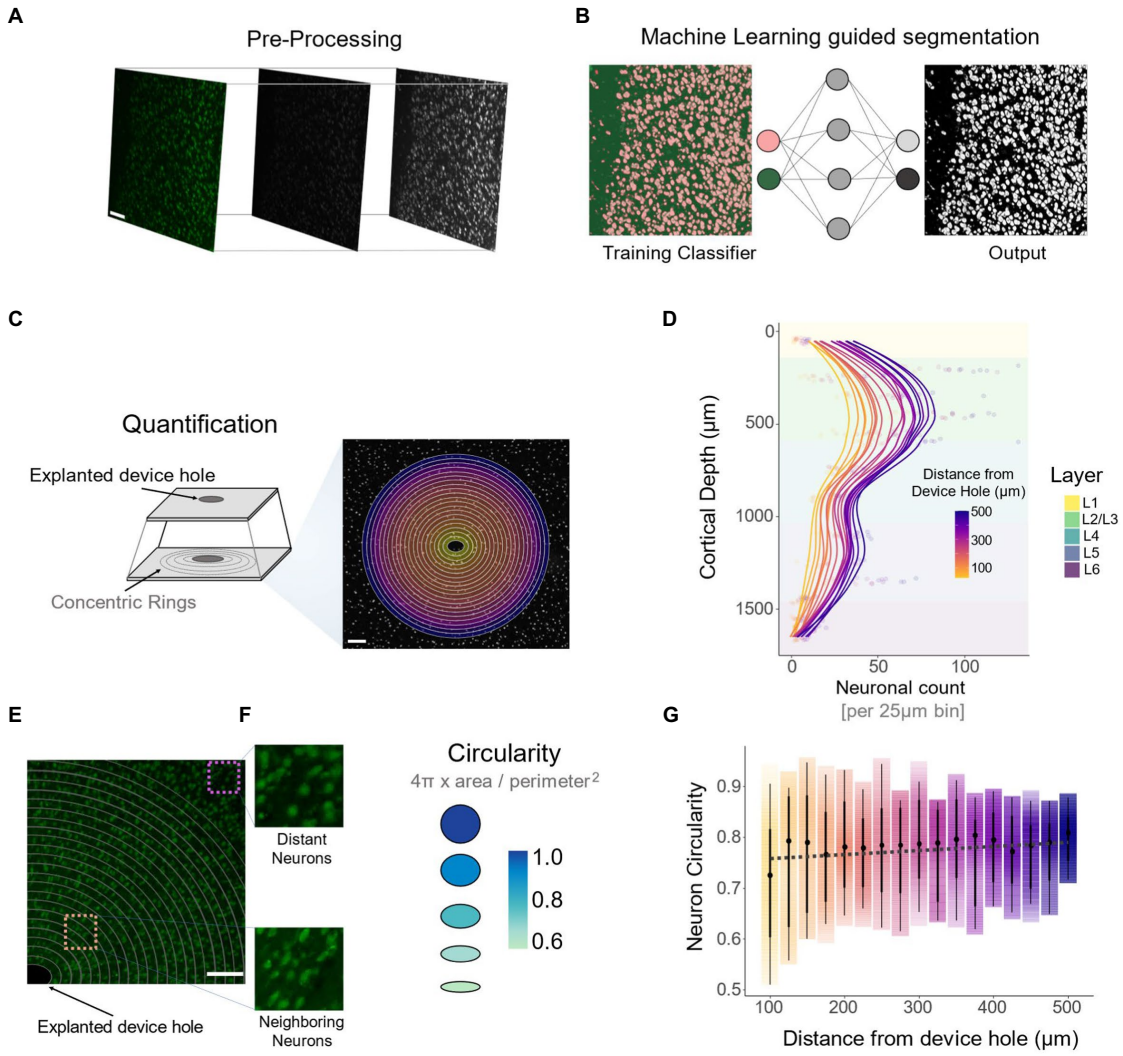
Quantification of neuronal density across cortical depth and cellular morphology *via* DeepHisto Machine learning segmentation. **(A)** Pre-processing pipeline consisting of 8-bit conversion followed by a Gaussian blur and contrast enhancement. **(B)** A multilayer perceptron machine learning classifier was trained to classify background pixels from individual neurons. **(C)** Following scaling, 25 μm isometric concentric rings were drawn around the device hole. **(D)** Number of individual neuronal nuclei per 25 μm bin across cortical depth (*N* = 5). **(E)** Representative image of morphological differences of neurons neighboring the electrode (bottom, orange square) compared to neurons away from the electrode (top purple square). **(F)** To assess morphological differences in neurons surrounding the device, circularity was computed. **(G)** Quantification of neuronal circularity and distance from the device hole. Black dots represent the median and thick lines represent 95% confidence intervals. The opacity of the color gradient represents data density. All scale bars are 100 μm.

We used machine learning (ML) to assess the relationship between neuronal cell loss (Figures 7C,D) and neuronal density across cortical depth. The ML segmentation algorithm (Figures 8A,B) quantified the number of neuronal nuclei on concentric rings radially diverging from the electrode hole (Figure 8C). Consistent with previous reports (Thelin et al., 2011; Du et al., 2017), the overall density of neurons peaks in L4. This is followed by a decrease in L5 and a secondary peak in L6 (Figure 8D).

Visual inspection of surviving neurons neighboring the explanted electrode hole revealed a change in morphology compared to distant neurons (Figure 8E). To quantify this, we computed the mean circularity of neurons within each concentric ring. Circularity is a measure of roundness based on the area-to-perimeter ratio, with a value of 1 representing a perfect circle (Figure 8F). The lower the value, the more elongated the object is (Figure 8F). The spread of confidence intervals and the mean circularity of neurons decrease as a function of proximity to the electrode (Figure 8G: −0.079 circularity units/mm, linear regression slope). In other words, cells neighboring the electrode are more elongated and have a higher degree of morphological variability compared to distant neurons. Moreover, the ratio of circularity between neighboring (100–200 μm from the electrode) and distant (400–500 μm from the electrode) neurons remained constant across cortical depth (Supplementary Figure S2: 0.92 ± 0.10 circularity units). By the deepest channels, however, this ratio was closer to 1, implying that neighboring neurons around the electrode tip were not as elongated (0.82 ± 0.13 circularity units) as in the other cortical depths (Supplementary Figure S2: 0.75 ± 0.10 circularity units). These findings indicate that compared to distant neurons, surviving neurons in proximity to the electrode are more elongated. However, this change in morphology disappears near the narrower tip of the electrode.

## Discussion

**Figure 9:**
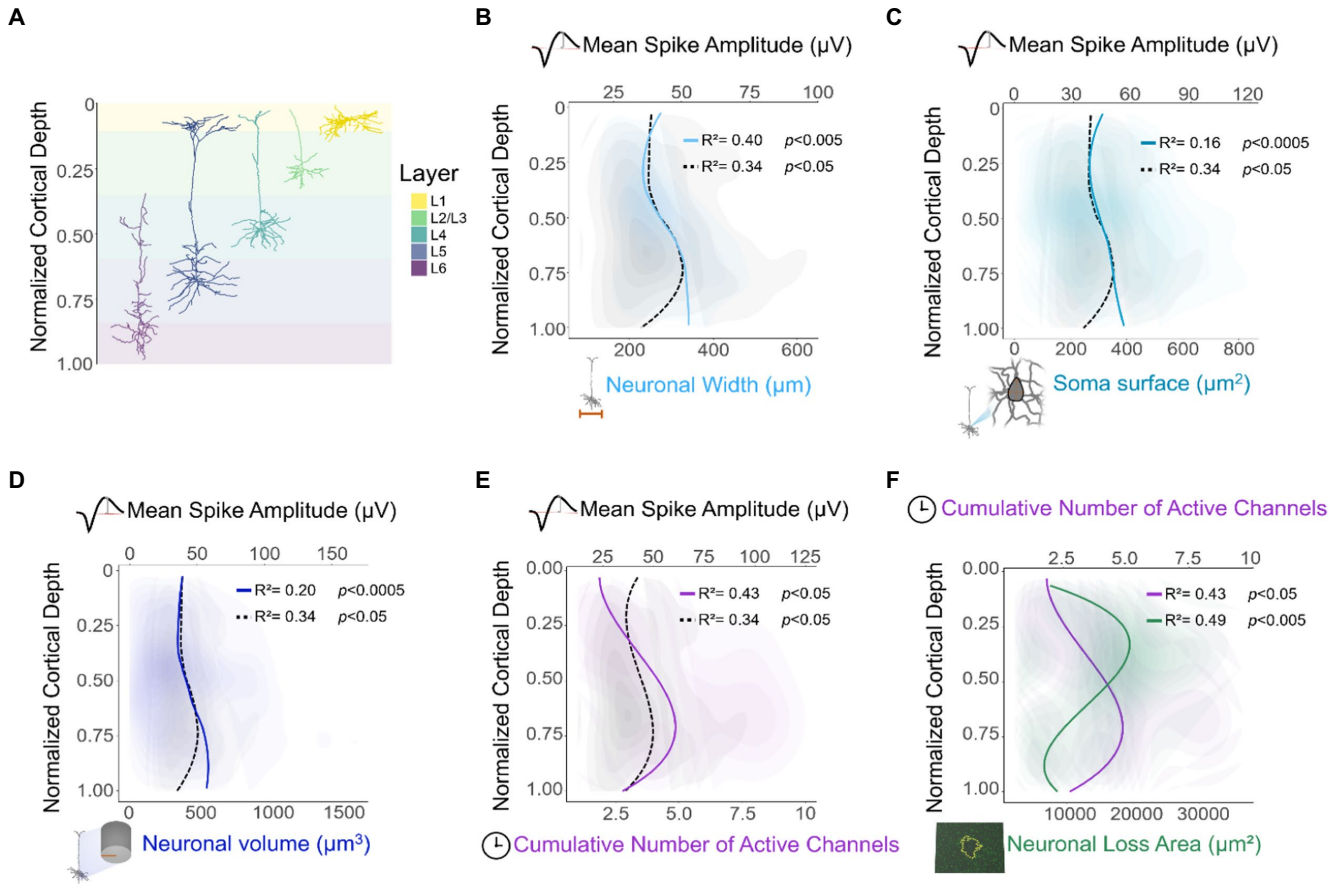
Relationship between histological, electrophysiological, and morphological features of neurons across cortical depth. **(A)** Representative diagram of individual neuronal reconstructions across cortical layers (Senzai et al., 2019). **(B–F)** Locally weighted scatterplots and locally fitted polynomial regressions were applied for visualization purposes. Additionally, a nonlinear correlation across cortical depth was computed *via* spatial sampling. To assess the relationship between depth-dependent recording patterns of this study and neuronal morphology, morphological features were extracted from 3D reconstructions of mouse cortical neurons (*n* = 513) (Senzai et al., 2019). Neuronal width **(B)**, soma surface area **(C)**, and volume of the entire neuron **(D)** were compared in relationship with the mean spike amplitude across normalized cortical depth (same data as Figure 2B). Cumulative number of channels with active spikes across cortical depth (same data as Figure 3B) and its relationship with **(E)** mean spike amplitude (same data as Figure 2B), and **(F)** neuronal cell loss (same data as Figure 7C).

Consistent with previous reports (Li and Jasper, 1953), recording performance could be related to layer-specific differences in neuronal morphology. Initial assessment of intracortical recordings across cortical depth showed a peak in spike waveform amplitude around L4-L5. This depth-related change in amplitude could be linked to the morphological heterogeneity (Gouwens et al., 2019) of neurons across cortical layers (Figure 9A). In the 1950’s, Li and Jasper (1953) recorded neurons from the somatosensory cortex of cats at different cortical depths. This group found that although most spikes had similar durations, L5 pyramidal cells had the highest spike amplitudes. This group also reported a robust relationship between spike amplitude and neuronal size (Li and Jasper, 1953). Additionally, neurons in L4-L5 have shown high-amplitude evoked excitatory synaptic responses in both humans and mice (Seeman et al., 2018). Given the canonical circuitry and broad similarities in cellular organization across cortical areas and species (Harris and Shepherd, 2015), we compared our results with neuronal morphological features of mouse visual cortex (Staining, 2015). Our analysis showed a robust depth-specific relationship between our recorded spike amplitude and neuronal width (Figure 9B), surface area of the soma (Figure 9C), and neuronal volume (Figure 9D). Moreover, neurons in L5 (specifically thick-tufted pyramidal cells) (Figure 9A, L5 neuron) tend to dominate the output of a cortical column (De Kock et al., 2007). Indeed, an increase in firing rate in L5 seems to be conserved across species (De Kock et al., 2007; Senzai et al., 2019), and cortical areas (Sakata and Harris, 2009; Gouwens et al., 2019; Ryu et al., 2019).

The long-term stability of spike amplitude, SNR, and active electrode sites varies in a layer-dependent manner. The mean recording amplitude increased during the first few weeks and remained stable throughout the rest of the study (Figure 4B), consistent with impedance (Prasad and Sanchez, 2012) results (Figure 5D). Previous studies have reported a slight increase in recording amplitude during the first weeks (Vetter et al., 2004; Guan et al., 2019) followed by a stabilization period for the first few months after implantation (Vetter et al., 2004; Jackson and Fetz, 2007; Kozai et al., 2016). Over years post-implantation, however, the amplitudes of the spikes tend to decay (Chestek et al., 2011; Barrese et al., 2016; Hughes et al., 2021). Our analysis showed that the stability of spike amplitude changes significantly across cortical layers, with L4-L5 spikes having the highest spike amplitudes throughout the study (Figures 2B,C). In contrast, using recordings in visual cortex from anesthetized mice, Kozai et al. (2016) found that the highest spike amplitudes gradually shifted from L4-L5 to L2/3 over time. On the other hand, our mean SNR decreased over time, consistent with previous studies (Vetter et al., 2004; Prasad and Sanchez, 2012; Barrese et al., 2016; Kozai et al., 2016; Hughes et al., 2021). However, the chronic SNR trend changed across cortical layers (Kozai et al., 2015), with L5 consistently having higher SNR values across the duration of the study. Moreover, previous studies have reported great variability in the overall number of active sites over time (Williams et al., 1999; Barrese et al., 2016). Here, this variability arose mostly from the cortical depth of the interfacing electrode, with electrodes-sites located in L4-L5 remaining active for longer periods of time. In the case of multielectrode arrays, it is possible that clusters of electrode sites may lay at different cortical depths due to the gyrification of the cortex, influencing the recording capabilities of contiguous electrodes (Riehle et al., 2018) and the their long-term performance.

A potential limitation of our results is that all of the electrodes in our study were subject to intracortical microstimulation (ICMS) experiments (Urdaneta et al., 2019; Kunigk et al., 2022; Urdaneta et al., 2022); it is unclear how this ICMS may have affected recording performance. Despite the lack of unstimulated electrodes as a control group, all microstimulation charges were distributed across all channels. Furthermore, the mean microstimulation charge was 9.3 ± 6.08 nC/Phase, a charge deemed safe and known to induce no significant tissue damage (Rajan et al., 2015). Hughes et al. (2021) found a similar decrease in recording stability for both stimulated and non-stimulated electrodes, with stimulated electrodes having higher spike amplitudes than non-stimulated electrodes. Moreover, there was no significant relationship between injected charge and signal quality (Hughes et al., 2021). These results suggest that ICMS at the levels applied in the current study does not affect the stability of intracortical recordings (Rousche and Normann, 1998; Parker et al., 2011; Ferroni et al., 2017; Hughes et al., 2021) and it may even contribute to recording improvements (Johnson et al., 2005; Otto et al., 2006).

Clinically relevant BMI technologies rely on decoding neural signals from the cortex over long periods of time after implantation to control computer cursors, robotic arms, and exoskeletons (Nicolelis, 2003). Once cortical activity is acquired (i.e., through an intracortical neuroprosthetic device), the decoding quality of these signals remains stable for multiple years (Pandarinath et al., 2018; Gallego et al., 2020). Nevertheless, as previously discussed, acquiring high quality neural signals over time can be challenging. A gradual decrease in SNR and number of active electrode sites might degrade BMI performance and its clinical translatability (Lebedev and Nicolelis, 2017). Given the laminar differences in long-term recording stability of single units, cortical depth can play a previously unexplored role in the stability of BMI technologies. Moreover, cortical depth also influences the acquisition of other types of neural signals used in BMI applications (Flint et al., 2016; Milekovic et al., 2018), such as multi-unit activity (MUA) (Kozai et al., 2015; Fiáth et al., 2016), and local field potentials (LFP) (De Kock et al., 2007; Kozai et al., 2015; Ryu et al., 2019; Senzai et al., 2019).

Cortical depth can play an important role in the type of information extracted for BMIs. BMI performance does not solely rely on the quality of the acquired signals. The type of features that can be extracted from these signals plays an important role in the quality of the decoding algorithm (Nicolelis, 2003). Sensory stimuli or intent can increase the firing frequency of neurons. This change in firing rate (FR) is a commonly used feature for BMI applications; for instance, neurons in the motor cortex can increase their FR prior to the movement of an arm in a particular (“preferred”) direction (Georgopoulos et al., 1982). Recordings from such a neuronal population can then be used to decode a preferred direction algorithm for a computer cursor or a robotic arm (Hochberg et al., 2012). The response properties of neurons vary across cortical depth (Gilbert, 1977), playing a role in the type of information that can be decoded for BMI applications. Hence, it is important to take interfacing depth into account in the design of cortical prostheses. For some cortical areas, it is possible to acquire neuronal signals at a single layer interface. For example, neurons that generate gaze onset and holding in the frontal eye fields (FEF) are exclusively located in L5 (Schall, 2008). However, it is more common for cortical areas to have more distributed neuronal encoding across cortical depth. For example, Chandrasekaran et al. showed a depth-dependent visuomotor response in the dorsal premotor cortex during a decision-making task (Chandrasekaran et al., 2017). They found that neurons that increase their FR during the task were more prevalent in superficial layers. Conversely, neurons that decreased their FR and neurons that responded to movement were mostly anatomically restricted to deeper layers (Chandrasekaran et al., 2017). A similar depth-specific neuronal response has been observed in the prefrontal cortex (PFC) during a discrimination task (Opris et al., 2011) and a working memory task (Markowitz et al., 2015). In the latter, the depth-related pattern of neuronal responses fluctuated even during different phases of the task (preparation, early, and late storage) (Markowitz et al., 2015). A similar depth-specific encoding has been observed during preparation, initiation, and execution of movement in the motor cortex (Isomura et al., 2009). Hence, recording neural signals across cortical depth can not only extend the long-term stability of BMI technologies, but also potentially improve the quality of decoding algorithms.

To evaluate the FBR across cortical layers, DeepHisto combines systematic cortical depth tracking (Kozai et al., 2014) with neuronal cell loss estimation, morphological assessment, and depth-specific quantification. The DeepHisto pipeline can be applied to a variety of FBR markers to obtain a clearer understanding of the biotic response to neuroprosthetic devices, especially for biocompatibility studies (such as those involving coatings) and novel electrode designs. In this study, however, DeepHisto was exclusively applied to depth-related patterns of neuronal density and morphology surrounding the electrode. Previous studies have reported neuronal cell loss around the explanted electrode (Biran et al., 2005; Salatino et al., 2017) as well as a change in morphology in surviving neurons (Thelin et al., 2011; Du et al., 2017). Our machine learning segmentation algorithm showed that neuronal deformation and variability increased as a function of electrode proximity and across cortical depth (Supplementary Figure S2). Further research is necessary to assess if this phenomenon is caused by biological changes in the cellular microenvironment (Du et al., 2017) or is a histological artifact caused by bending of the tissue.

One potential limitation of our recording performance and histological analysis across cortical depth is the geometry of the probe used in these studies. The width of the probe narrows monotonically with cortical depth. Previous studies in the literature have demonstrated that smaller probe geometries exert a reduced biological footprint and are characterized by eliciting a reduced FBR (Luan et al., 2017). Thus, the variance of probe substrate width in our studies confounds the observation of neuronal loss with cortical depth. However, our findings show that neuronal cell loss patterns do not follow a monotonic decay across depth. Given that the probe used in this experiment tapers down monotonically as a function of depth, a monotonic response of neuronal cell loss would be expected. In contrast, our findings showed a greater extent of neuronal cell loss around lower L2/3 and upper L4 compared to other depths. These results are consistent with the greater extent of neuronal cell loss observed in histological sections of mid-cortex compared to superficial and deep sections (Rosenfeld et al., 2020). Given that spike amplitude decays as a function of distance from the electrode (Buzsáki et al., 2012) and that noise floor increases over time (Vetter et al., 2004), it is possible that the spike amplitude of surviving neurons might not be large enough to be detected by the electrode above the noise level. This could, in part, explain the inverse relationship between the cumulative number of active sites (Figure 9E) and the area of neuronal cell loss across cortical depth (Figure 9F).

Disruption of vasculature may lead to an exacerbated neuronal cell loss across layers. Device insertion and constant micromotion from a stiff material can cause changes in the cellular microenvironment that can lead to the loss of neurons surrounding the electrode (Du et al., 2017). Furthermore, the density of neurons and blood vessels can potentially exacerbate the area of neuronal cell loss. Consistent with previous reports in the somatosensory cortex of naïve rats (Meyer et al., 2013), our quantification of neuronal nuclei showed density peaks in L4 and L6, with a steep decrease around L5. Similarly, experiments in mice have shown that the distribution of blood vessels fluctuates across depth (Blinder et al., 2013; Guy et al., 2015), with a peak around lower L2/3 and upper L4 (Guy et al., 2015). This pattern considerably resembles the profile of neuronal cell loss reported here. Even though major vessels are avoided during device implantation, the disruption of microvasculature is unavoidable. The mechanical mismatch of the implanted electrode and surrounding tissue (Du et al., 2017) can lead to vessel disruption and loss of perfusion of neuronal cells necessary for the proper functioning and health of neurons.

In conclusion, we report increased quality of longitudinal intracortical recordings around L4 and L5 compared to other layers, which may be related to morphological differences across cortical layers. Chronic indicators of recording performance such as SNR, number of active sites, and spike amplitude changed based on the interfacing cortical depth, with electrode sites located in L4-L5 exhibiting the highest long-term stability. To assess the biological response of these chronically implanted electrodes across cortical depth, we developed DeepHisto. This technique revealed that the extent of neuronal cell loss changes across cortical depth and is exacerbated around L2/3 and L4. Moreover, our machine learning-guided segmentation revealed morphological changes in surviving neurons neighboring the electrode. These findings expand on the previous literature by systematically investigating both recording performance and the foreign body response in a layer-specific manner. Altogether, our findings suggest that cortical depth may play a previously under-explored role in the development of intracortical electrodes and the long-term stability of BMI technologies.

## Methods

### Surgical procedure

All animal experiments and surgeries were performed under the approval and guidance of the Institutional Animal Care and Use Committee (IACUC) of the University of Florida (Gainesville, FL, United States). Surgeries were performed by the same surgeon using aseptic techniques. Prior to implantation, the silicon microelectrode device was gas sterilized with ethylene oxide and rinsed with sterile saline. This device had 16 iridium electrode sites (703 μm^2^) spanning all layers of the rodent cortex (A1 × 16-3 mm-100-703-HZ16, NeuroNexus, Ann Arbor, MI) (Figure 1A). The spacing between electrode sites was 100 μm. Six adult male Sprague–Dawley rats (450–650 g, Charles River, Chicago, IL, United States) were given meloxicam (1–2 mg/kg, SQ, Loxicom, Norbrook Laboratories, Newry, Northern Ireland) then induced at 5% and maintained at 1.5–3% isoflurane (Zoetis, Parsippany, NJ, United States) in oxygen at 1.5–2 l/min. Following skin incision and periosteum removal, a 1 mm^2^ cranial window was prepared over the primary somatosensory cortex using a microdrill. Four burr holes were drilled to secure titanium bone screws (United Titanium, OH) for grounding and headcap anchoring. The implantation target was the forepaw region of the right primary somatosensory cortex [0.5 mm anterior to bregma, 3.5 mm lateral to midline (Paxinos and Watson, 2006)]. Following a dural slit, an automated micro-insertion system (PiLine M663, Physik Instrumente, Karlsruhe, Germany) was used to insert the microelectrode device 1,600 μm from the cortical surface at 10 mm/s. Implantation depth was verified by assessing complete insertion of the most superficial electrode using a surgical microscope. After insertion, the craniotomy site was filled with silicon elastomer (Kwik-Sil, WPI, Sarasota, FL) followed by layers of UV-cured dental composite (DentalSource, CA) to secure the electrode and anchor the headstage connectors (Saldanha et al., 2021).

### Neural recordings and impedance measurements

All electrophysiological recordings and impedance measurements were obtained from awake, freely behaving animals. Animals were habituated in a custom-made plexiglass box inside a Faraday cage prior to surgery. This habituation period was critical to reduce movement artifacts during recordings. Neural recordings from all 16 channels were taken at a sampling rate of 24,414 Hz *via* a PZ5 NeuroDigitizer amplifier (Tucker Davis Technologies, Alachua, FL) connected to an RZ5D Bioamp processor (Tucker Davis Technologies, Alachua, FL). Weekly 5 min recording sessions were obtained during the first month post-implantation, and twice a month afterwards up to 16 WPI. Once a recording session was over, the animal was connected to a passive ZIF-Clip headstage (ZC32P, Tucker Davis Technologies, Alachua, FL) to obtain electrochemical impedance spectroscopy (EIS) measurements. Logarithmic frequency sweeps from 10 Hz to 100 kHz were measured using a PGSTAT128N Potentiostat (Metrohm Autolab, Utrecht, Netherlands). Each frequency sweep was conducted three times and averaged. All EIS measurements had a potential of 15 mV peak-to-peak and 0 V DC bias. The animals were also used in ICMS experiments. These experiments were conducted 3–5 days a week. To avoid changes in impedance due to microstimulation, all recordings and impedance measurements preceded ICMS experiments during an experimental session. All ICMS charges were limited to 30nC/Phase and were distributed across all channels. To avoid damage and extend the life of the connectors, a custom-made metallic protective cap was fit over the headcap after each session.

### Neural recordings analysis

Raw neural signals were band pass filtered at 250 Hz – 3 kHz using a 1st order Butterworth filter in MATLAB (MathWorks, Natick, MA). Even though, recent work has shown that there is valuable information in high frequency signals (RaviPrakash et al., 2021), band-pass filtering, as the one used herein, has been a commonly used technique for spike sorting (Quiroga, 2012). Spike sorting analysis was performed using Offline Sorter (V3, Plexon Inc. Dallas, TX). High amplitude artifacts (>90% peak amplitude) simultaneously occurring across multiple channels were invalidated. For spike detection, a threshold of four times the noise level σN (Equation 1) was used.


(1) $$\sigma N=\frac{\mathrm{median}\left(\mathrm{abs}\left(\mathrm{signal}\right)\right)}{0.675}$$

An automatic K-means clustering algorithm (Offline Sorter K-Means Scan) was applied to the principal component domain to sort single units (Figure 1B; Plexon Offline Sorter Manual, n.d.). Sorted units were visually inspected for each channel by a blinded evaluator. The signal-to-noise ratio was calculated for each channel by dividing the peak-to-peak amplitude over two times the noise level (Equation 2).


(2) $$SNR=\frac{\mu_{\mathrm{pp}}}{2\sigma_{N}}$$

Only spike waveforms that passed the evaluator’s visual inspection and had more than 200 single units were considered for analysis. Waveform amplitudes (Figure 3A) were extracted using NeuroExplorer (V4, Nex Technologies, CO) by calculating the average amplitude of all spikes.

### Implantation depth assessment

An air puff onset was directed at the animal’s contralateral forepaw during electrophysiological recordings. The air puff system was fabricated in-house and was triggered *via* a 5 V TTL pulse from a RZ5D Bioamp processor (Tucker Davis Technologies, Alachua, FL). The voltage traces following the air puff stimulus were averaged and plotted across depth. These traces were used to calculate the inverse current source density (iCSD) (Pettersen et al., 2006; Supplementary Figure S1A). Histological verification of electrode location was assessed *via* an electrolytic DC lesion (50 μA anodic pulse for 1 s) (Supplementary Figure S1B).

### Tissue processing

A serial tissue slice acquisition scheme with sections evenly dispersed among sequential slides was used to track cortical depth. Following immunohistochemistry for neuronal nuclei, images were captured using fluorescence microscopy and quantification of neurons was conducted at known depths along the implant track.

### Tissue collection

After the culmination of recordings and EIS measurements at 16 WPI, the animals were continually used for ICMS experiments. At the conclusion of the microstimulation experiments, animals were induced in 5% isoflurane (Zoetis, Parsippany, NJ) in oxygen at (2 l/min). A transcardial perfusion was performed using phosphate buffered saline (PBS) followed by 4% paraformaldehyde (PFA) solution. Perfusions were performed on all animals between 27 and 42 WPI except for one animal that died of natural causes at 35 WPI. The skulls of perfused and non-perfused animals were incubated in 4% PFA at 4°C for 72 h. Following multiple washes in PBS, the skulls were removed and the devices carefully explanted (Figure 6A: step 1). The extracted brains were cryoprotected at 30% sucrose in PBS for 72 h at 4°C and immediately flash frozen at − 40°C in 2-methylbutane (Sigma Aldrich, St. Louis, MO).

### Cryosectioning to track cortical depth

A core of brain tissue with a ~ 5 mm radius around the implanted device of cortex surrounding the explanted device was resected and mounted on a cryosection chuck with Tissue-Plus O.C.T. Compound (23–730-571, Fisher). Serial 20 μm tissue sections, sliced with a cryostat (CM 1520, Leica) at −20°C, were taken tangential to the cortical surface and adhered to SuperFrost Plus microscope slides (12–550-15, Fisher), with each new slice being added to the next in sequence, for a total of 8 slices per slide. Therefore, the first tissue slice on slide 1 represents a cortical depth of 0 to 20 μm, the first slice on slide 2 corresponds to cortical depth 20–40 μm, and so on. This was repeated in cycles for all slides to track tissue depth by slide identity and slice position (Figure 6B). Sectioned tissues were kept in −20°C until used for staining.

### Immunohistochemistry

Immunohistochemistry (IHC) was performed to identify neuronal nuclei using Anti-NeuN (ABN90, EMD Millipore) and FITC anti-guinea pig (706–095-148, Jackson ImmunoResearch Laboratories Inc.) (Figure 6C). Primary and secondary antibodies were diluted to 1:500 with Animal-free Blocker and Diluent, R.T.U. (SP-5035, Vector Laboratories Inc.). Slides were kept at room temperature (RT) for 1 h, then washed for three 5 min cycles in PBS with gentle rocking. Next, slides were incubated in R.T.U. buffer for 12 h at 4°C. Following removal of excess buffer, the diluted primary antibodies were distributed between slides and incubated at 4°C for 36 h. Following three 10 min PBS wash cycles, slides were stained with secondary antibodies, diluted and distributed same as before, then incubated at 4°C for 24 h. After three 10 min PBS washes excess liquid was aspirated and glass coverslips were mounted using VectorShield medium with DAPI (H-1200, Vector Labs). Slides were kept in the dark at RT for 24 h before imaging.

### Fluorescence microscopy

Image acquisition was performed on a Leica DMi8 microscope running LAS X Premium software. Peripheral components include a DFC9000 GT camera, EL 6000 light source (30 ms), HC PL APO 10x/0.45 objective, and GFP filter cube. Acquisitions were centered over the implant scar.

### Neuronal cell loss estimation

Quantification of neuronal cell loss area was performed by manually tracing the area surrounding the explanted hole with no evident neuronal bodies (Figure 6D). This assessment was performed by two blinded evaluators using a custom-made script in Fiji (Schindelin et al., 2012; Figure 7A). The neuronal cell loss area measured by each evaluator was then averaged and analyzed across cortical depth for each animal (Figure 6E).

### Machine learning

Images were pre-processing with a grayscale conversion, followed by a 0.2% contrast enhancement and application of a dynamic gaussian Blur (Figure 8A; Schindelin et al., 2012). Based on shape and intensity, pixels from neuronal nuclei and background were classified using a Trainable Weka Segmentation tool (Arganda-Carreras et al., 2017). Roughly 5% of all histological sections at random depths were chosen as the training dataset. These were trained on a Multilayer Perceptron (MLP) classifier with default parameters (learning rate: 0.3, momentum: 0.2) and features (Arganda-Carreras et al., 2017). The main goal of using machine learning guided segmentation was to improve the performance of available auto-thresholding methods (Huang, Li, Mean, intermodes, etc.) during segmentation. Following training (Malone et al., 2022), the image segmentation results were assessed by the expert opinion of two experimenters to assess if these were more accurate than any available auto-thresholding method. Post-segmentation, a series of 25 μm concentric rings around the explanted device hole were drawn using a custom-made script in Fiji (Schindelin et al., 2012). The number of neuronal nuclei and the mean circularity (Figure 8F) of each concentric ring was computed and analyzed across cortical depth.

### Morphological correlations

A total of 513 neuronal reconstructions from the Allen cell types database (Staining, 2015) were analyzed based on their normalized cortical depth. These neurons were predominantly from the primary visual area (87.1%) of the mouse. Morphological features such as overall width (Figure 9B), soma surface area (Figure 9C), and overall volume (Figure 9D) were used for analytical comparison across normalized cortical depth. Min-max normalization was used to normalize the depth of mice and rat cortices.

### Statistical analysis

Statistical analyses were performed in R Statistical Software Version 4.0.0 (Vienna, Austria). Normality was tested using a Shapiro-Wilks test. Similarly, assumptions for homogeneity of variance between groups were confirmed using Levene’s test. For parametric comparisons (Figures 2C, 3B, 4C,D,G,H, 7D), significant differences were determined *via* one-way ANOVA followed by Tukey’s *post hoc* test. Pairwise comparisons across cortical layers were assessed using a two-tailed unpaired t-test; to correct for multiple comparisons, a Dunn-Bonferroni’s adjustment was applied. For non-parametric comparisons (Figures 5B,C), significant differences were determined using a Kruskal–Wallis test. Pairwise comparisons across days were determined using Wilcoxon rank–sum tests. *p ≤ 0.05, **p ≤ 0.01, ***p ≤ 0.001, ****p ≤ 0.0001.

## Data availability statement

The original contributions presented in the study are included in the article/Supplementary material, further inquiries can be directed to the corresponding author.

## Ethics statement

The animal study was reviewed and approved by all animal experiments and surgeries were performed under the approval and guidance of the Institutional Animal Care and Use Committee (IACUC) of the University of Florida (Gainesville, FL).

## Author contributions

MU, NK, JP-A, SC, IM, SF, and KO conceived and designed the study and reviewed and edited the manuscript. MU performed the surgical implantations. MU and NK collected the data. MU, NK, JP-A, SC, and IM contributed to process and analyze the data. All authors contributed to the article and approved the submitted version.

## Funding

This work was sponsored by the NIH (NIH-U01NS099700 and NIH-1OT2OD023861–01).

## Conflict of interest

The authors declare that the research was conducted in the absence of any commercial or financial relationships that could be construed as a potential conflict of interest.

The reviewer MD declared a shared affiliation with the author SF to the handling editor at the time of review.

## Publisher’s note

All claims expressed in this article are solely those of the authors and do not necessarily represent those of their affiliated organizations, or those of the publisher, the editors and the reviewers. Any product that may be evaluated in this article, or claim that may be made by its manufacturer, is not guaranteed or endorsed by the publisher.
