# Supplementary material for: Layer-dependent stability of intracortical recordings and neuronal cell loss
Source: Front Neurosci. 2023 Apr 5;17:1096097. doi: 10.3389/fnins.2023.1096097 (PMC10113640; doi:10.3389/fnins.2023.1096097)
Supplement: Supplementary file 1 [file Data_Sheet_1.docx]

**Supplementary Material**


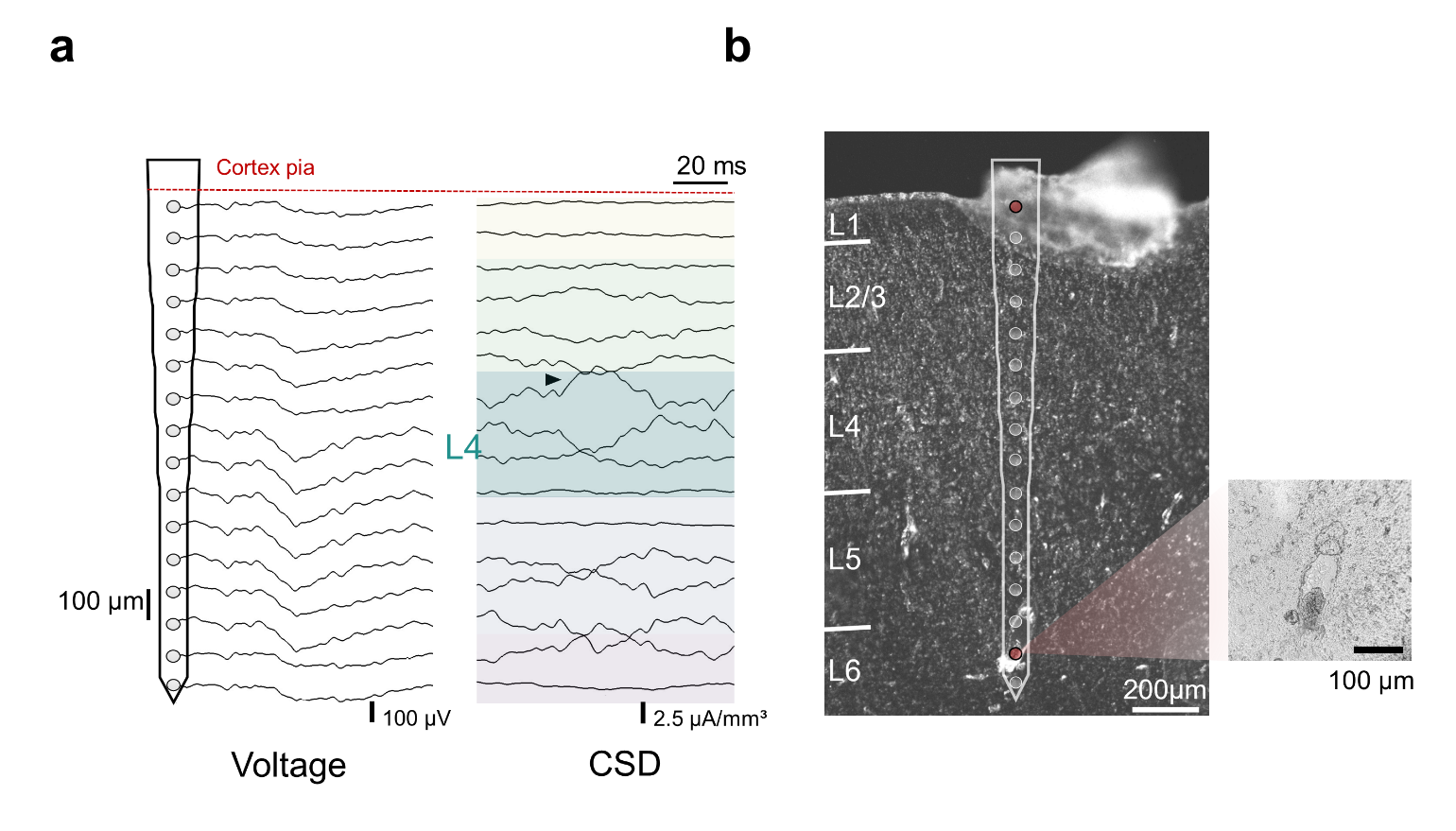


**Supplemental Figure 1. Electrophysiological and post-mortem implantation depth assessment.** a) Voltage traces and inverse current source density (iCSD) profile following an air puff onset to the animal’s contralateral forepaw. Black arrow indicates a current sink around 650 µm from the cortical surface. This initial sink, putative of thalamic input, is characteristic of upper layer 4^32^. b) Histological verification of electrode location following electrolytic DC lesion of channels (red electrode-sites).


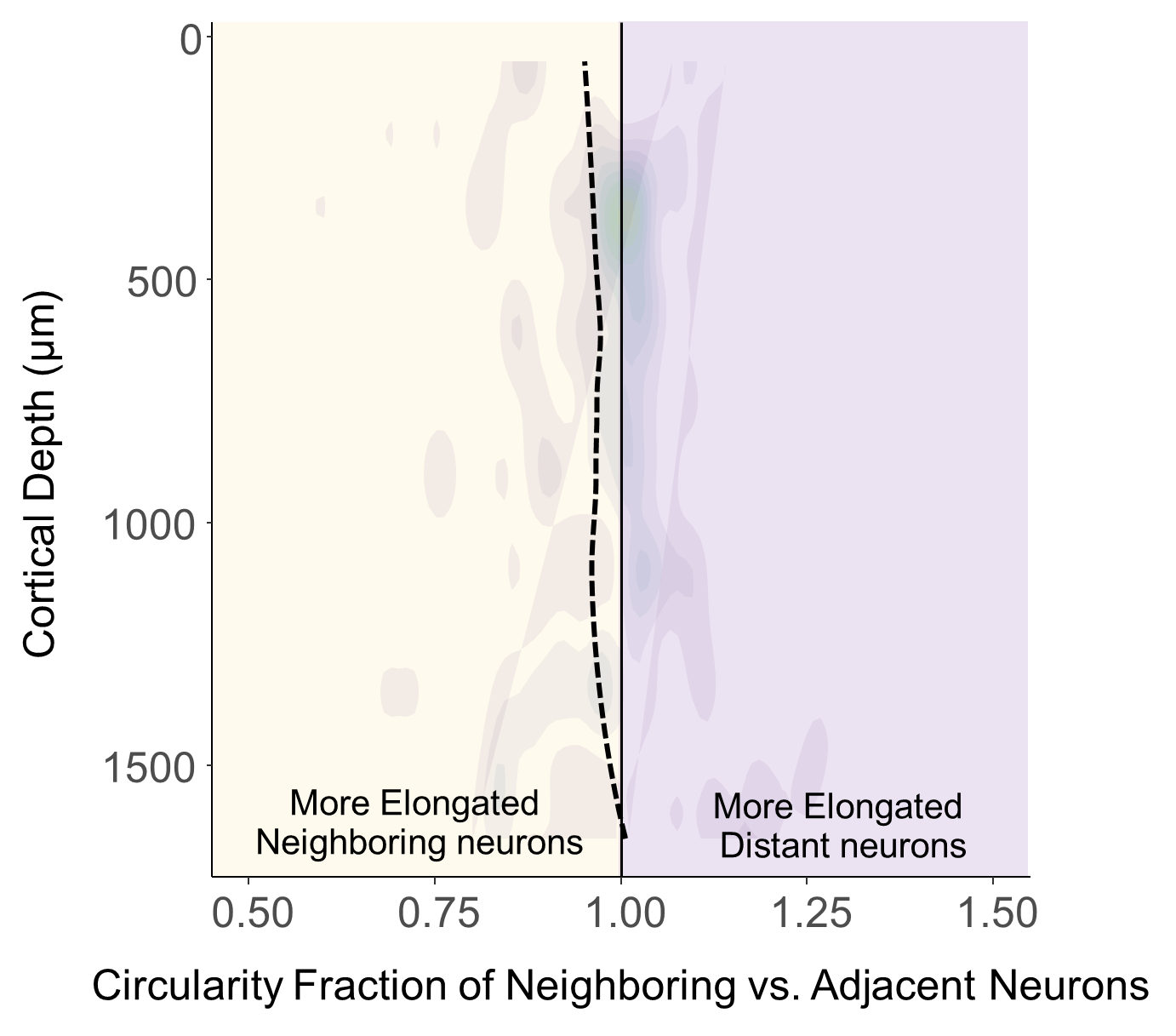


**Supplemental Figure 2. Neuronal circularity across cortical depth.** The circularity ratio of neighboring (100-200 µm from the electrode) and distant (400-500 µm from the electrode) neurons. Values below 1 indicate that neurons are more elongated than distant neurons. Dashed line represents a locally fitted polynomial regression of the means and shaded regions represent a locally weighted scatterplot (2D Kernel Density) of circularity values (N = 5).
